# Degradation of Recalcitrant Polyurethane and Xenobiotic Additives by a Selected Landfill Microbial Community and Its Biodegradative Potential Revealed by Proximity Ligation-Based Metagenomic Analysis

**DOI:** 10.3389/fmicb.2019.02986

**Published:** 2020-01-22

**Authors:** Itzel Gaytán, Ayixon Sánchez-Reyes, Manuel Burelo, Martín Vargas-Suárez, Ivan Liachko, Maximilian Press, Shawn Sullivan, M. Javier Cruz-Gómez, Herminia Loza-Tavera

**Affiliations:** ^1^Departamento de Bioquímica, Facultad de Química, Universidad Nacional Autónoma de México, Mexico City, Mexico; ^2^Departamento de Química Analítica, Facultad de Química, Universidad Nacional Autónoma de México, Mexico City, Mexico; ^3^Phase Genomics Inc., Seattle, WA, United States; ^4^Departamento de Ingeniería Química, Facultad de Química, Universidad Nacional Autónoma de México, Mexico City, Mexico

**Keywords:** biodegradation, microbial community, polyether-polyurethane-acrylate, xenobiotic additives, metagenomics, Hi-C proximity-ligation, community structure, biodegradative potential

## Abstract

Polyurethanes (PU) are the sixth most produced plastics with around 18-million tons in 2016, but since they are not recyclable, they are burned or landfilled, generating damage to human health and ecosystems. To elucidate the mechanisms that landfill microbial communities perform to attack recalcitrant PU plastics, we studied the degradative activity of a mixed microbial culture, selected from a municipal landfill by its capability to grow in a water PU dispersion (WPUD) as the only carbon source, as a model for the BP8 landfill microbial community. The WPUD contains a polyether-polyurethane-acrylate (PE-PU-A) copolymer and xenobiotic additives (*N*-methylpyrrolidone, isopropanol and glycol ethers). To identify the changes that the BP8 microbial community culture generates to the WPUD additives and copolymer, we performed chemical and physical analyses of the biodegradation process during 25 days of cultivation. These analyses included Nuclear magnetic resonance, Fourier transform infrared spectroscopy, Thermogravimetry, Differential scanning calorimetry, Gel permeation chromatography, and Gas chromatography coupled to mass spectrometry techniques. Moreover, for revealing the BP8 community structure and its genetically encoded potential biodegradative capability we also performed a proximity ligation-based metagenomic analysis. The additives present in the WPUD were consumed early whereas the copolymer was cleaved throughout the 25-days of incubation. The analysis of the biodegradation process and the identified biodegradation products showed that BP8 cleaves esters, C-C, and the recalcitrant aromatic urethanes and ether groups by hydrolytic and oxidative mechanisms, both in the soft and the hard segments of the copolymer. The proximity ligation-based metagenomic analysis allowed the reconstruction of five genomes, three of them from novel species. In the metagenome, genes encoding known enzymes, and putative enzymes and metabolic pathways accounting for the biodegradative activity of the BP8 community over the additives and PE-PU-A copolymer were identified. This is the first study revealing the genetically encoded potential biodegradative capability of a microbial community selected from a landfill, that thrives within a WPUD system and shows potential for bioremediation of polyurethane- and xenobiotic additives-contamitated sites.

## Introduction

Plastic pollution represents a pervasive anthropogenic threat for the survival of natural ecosystems. Worldwide, plastics have become so abundant that they have been proposed as geological markers for the Anthropocene era (Zalasiewicz et al., 2016). In 2017, a total of 348 million tons of plastics were manufactured (Plastics Europe, 2018) and their production keeps increasing. Polyurethanes (PU) are versatile plastics produced as thermoplastics, thermosets, coatings, adhesives, sealants and elastomers that are incorporated into our daily life in building insulation, refrigerators and freezers, furniture and bedding, footwear, automotive, clothing, coatings, adhesives, and others. PU was ranked as the sixth most used polymer worldwide with a production of 18 million tons in 2016 (Cornille et al., 2017). The extensive utilization of PU generates wastes that are mainly disposed in municipal landfills where, because of its structural complexity will remain as polymeric structures for decades, or are burned generating toxic substances that negatively impact human health and ecosystems (Cornille et al., 2017). Furthermore, some PU such as polyether (PE)-PU are more recalcitrant than others, and additionally, some PU-based liquid formulations contain additives that include secondary alcohols and glycol ethers that function as solvents or coalescent agents. Glycol ethers enter the environment in substantial quantities, are toxic for many microbial species (Kawai, 2010; Varsha et al., 2011; Malla et al., 2018) and represent a potential hazard for human health (Organization for Economic Co-operation and Development, 2003).

Over the last three decades, several research groups have isolated microorganisms capable of attacking PU (Oceguera-Cervantes et al., 2007; Cregut et al., 2013; Álvarez-Barragán et al., 2016; Gamerith et al., 2016; Osman et al., 2018; Magnin et al., 2019) and degrading xenobiotic additives (Ojo, 2007; Varsha et al., 2011). Also, the degradation capabilities of several fungal and bacterial communities over PU in compost, soil or liquid cultures (Zafar et al., 2014; Shah et al., 2016; Vargas-Suárez et al., 2019), and over some xenobiotics in different activated sludges (Ferrero et al., 2018) have been assessed. However, PU biodegradation is still a challenge for environmental and biological disciplines and little is known about structure or potential degradative enzymatic pathways of microbial communities capable of PU biodegradation. Metagenomics provides access to the structure and genetic potential of microbial communities, helping to understand the ecophysiological relationships governing the dynamics of their populations in the environment. Recently, a new approach has been developed that allows the reconstruction of individual genomes of microbial species using physical interactions between sequences within cells (Burton et al., 2014). This approach involves Hi-C proximity ligation and yields direct evidence of sequences co-occurrence within a genome. It is used for *de novo* assembly, identification of complete and novel genomes (Press et al., 2017) and for testing functional and phylogenetic hypotheses, surpassing other methods for clustering contigs by taxonomic origins (Wu et al., 2014; Breitwieser et al., 2019; Shaiber and Eren, 2019).

To characterize the biodegradation process of the recalcitrant plastic PE-PU by microbial communities, we adopted the commercial water PU dispersion PolyLack^®^ (Sayer Lack, México) that contains a proprietary aromatic polyether-polyurethane-acrylate (PE-PU-A) copolymer and the xenobiotic additives *N*-methylpyrrolidone (NMP), isopropanol (IP) 2-butoxyethanol (2-BE), dipropyleneglycol butyl ether (DPGB), and dipropyleneglycol methyl ether (DPGM). In this work, we provide comprehensive chemical and physical evidences of the capacity of a selected landfill microbial community to degrade an aromatic PE-PU-A copolymer and the aforementioned xenobiotic additives. In addition, we analyzed the structure and phenotypic potential of this community by applying the Hi-C proximity ligation technology. Based on these analyses, we identified a novel microbial landscape that can deal with PE-PU-A and xenobiotics additives degradation and proposed putative metabolic pathways and genes that can account for these capabilities. This is one of the few studies that combine physical and chemical analyses with metagenomics to elucidate possible metabolic pathways involved in xenobiotics biodegradation. Furthermore, this is the first metagenomic analysis of a polyurethane-degrading enriched landfill community. Understanding these pathways will help to design environmental biotechnological strategies that contribute to mitigate plastics and xenobiotics pollution and to achieve a better environmental quality.

## Materials and Methods

### Site Location and Sampling Procedure

Deteriorated PU foam samples were collected at El Bordo Poniente (BP) landfill, located at Nezahualcóyotl Estado de México, México (19°27′10′′N; 99°0′58′′W). The samples were visually identified in piles of waste and in lixiviate waters, gathered by hand with sterile latex gloves and placed in sterile plastic bags for transportation to the laboratory. Then, they were immediately used to start the enrichment cultures.

### Microbiological Techniques

For obtaining the enriched microbial community from the BP8 sample, approximately 1 cm^3^ was cut over a sterile Petri dish using a sterile scalpel. This PU foam piece was inoculated in 125 mL Erlenmeyer flask with 25 mL of minimal medium (MM) (Nakajima-Kambe et al., 1995) containing PolyLack^®^ (0.3% v/v), as the sole carbon source (MM-PolyLack). PolyLack^®^ Aqua Brillante (Sayer Lack, Prod. Num. UB-0800, México), mainly used for coating of wood floors with moderate transit, contains a proprietary aromatic PE-PU-A copolymer (≤30% w/v), and the additives NMP (≤6% v/v), 2-BE (≤5% v/v), IP (≤3% v/v), DPGB (≤2% v/v), DPGM (≤1% v/v), and silica (≤3% w/v) (Sayer Lack. Hoja de Datos de Seguridad de Materiales. PolyLack^®^ Aqua Brillante, UB-0800. 09.18.2014. Version 4/0. México). The flask was incubated at 30°C and 220 rpm for 7 days. Two ml of this culture were transferred to 25 ml of fresh MM-PolyLack and cultured for 7 days, repeating two more times, for a total of 28 days, before being conserved in MM-PolyLack glycerol 30% at −70°C. The microbial mixed culture obtained from the landfill community, named as BP8, was propagated and conserved by inoculating 25 ml of fresh MM-PolyLack with a 500 μl glycerol from the original enriched community, incubated for 7 days and used to prepare new glycerols. BP8 growth was quantified by dry weight. For that, flasks with MM-PolyLack (25 ml) were inoculated with fresh cells (3 mg/ml) harvested from pre-cultures grown in MM-PolyLack for 48 h at 37°C, 220 rpm. At different incubation times, cells of one flask were harvested, washed three times with phosphate buffer (50 mM, pH 7) and dried to constant weight.

### Cell-Substrate Interactions Techniques

For Cell surface hydrophobicity (CSH) measurements, cells were washed twice and suspended in phosphate buffer (0.05 M, pH 7) to an optical density (OD) of 0.6 (600 nm). The mixture of cell suspension (2 ml) and *n*-hexadecane was vortexed for 3 min and after that, the organic and aqueous phases were allowed to separate for 30 min. OD was measured in the cell suspension (aqueous phase). CSH is expressed as the percentage of adherence to hexadecane and it was calculated as follows: 100 × [1-(OD_600_ of the cell suspension after 30 min)/(OD_600_ of the initial cell suspension)] (Rosenberg et al., 1980). Emulsification capacity of the culture medium was determined by mixing 2 ml of cell-free supernatant (CFS) and 3 ml of *n*-hexadecane in glass test tubes. The tubes were vigorously vortexed for 2 min and afterward let to stand at room temperature. The emulsification index (EI_24_) was calculated after 24 h as follows: 100 × [emulsified layer height/total liquid column height] (Cooper and Goldenberg, 1987). To observe cell-copolymer interactions, cells were fixed with 3% (v/v) glutaraldehyde in phosphate buffer (100 mM, pH 7.4), at 4°C overnight, washed three times, dehydrated with serial dilutions of ethanol, coated with gold and analyzed in a JEOL JSM-5900-LV electron microscope.

### Analytical Techniques

Nuclear magnetic resonance (NMR) spectra from dried PolyLack^®^ dissolved in C_5_D_5_N (30 mg/ml) were recorded at 298 K in a Bruker Avance 400 NMR (Billerica, MA, United States) at 400 MHz (^1^H). For most of the analytical techniques, CFS were obtained by centrifugation at 17,211 × *g* for 10 min, filtered through Whatman grade 41 paper, and dried at 37°C for 5 days. Carbon content was determined in a Perkin Elmer Elemental Analyzer (2400 CHN/O, Series II, Shelton, CT, United States). For gas chromatography coupled to mass spectrometry (GC-MS) analysis, 25 ml CFS were extracted in 6 ml LC-18 cartridges (Supelco) at a flow rate of 2 ml/min, eluted with 2 ml chloroform:methanol (1:1, v/v) and concentrated to 0.5 ml. Samples were injected in an Agilent GC system (7890B, Santa Clara, CA, United States) using two 5% phenyl-methylpolysiloxane columns (15 m × 250 μm × 0.25 μm). Oven was heated from 50 to 300°C at 20°C/min, Helium was used as carrier gas at a flow rate of 1 ml/min. The injector temperature was 300°C. For the quantification of additives, pure compounds (Sigma-Aldrich Chemicals ≥98% purity) were used for standard curves. Identification of biodegradation products was performed in an Agilent Quadrupole Mass Analyzer (5977A MSD, Santa Clara, CA, United States) with electronic ionization energy of 1459 EMV and the mass range scanned at 30–550 amu. Scan rate was 2.8 spec/s. Data acquisition was performed with the Enhanced MassHunter software system. Compounds were identified based on mass spectra compared to the NIST database (2002 Library). Fourier transform infrared spectroscopy (FTIR) analyses were performed in dried CFS, by using a Perkin Elmer spectrometer (Spectrum 400, Waltham, MA, United States) in attenuated total reflection mode; 64 scans with a resolution of 4 cm^–1^ were averaged in the range of 500–4000 cm^–1^, processed and analyzed (Spectrum v6.3.5.0176 software). Derivative thermogravimetric analyses (DTG) were performed in a Perkin Elmer Thermogravimetric Analyzer (TGA 4000, Waltham, MA, United States) on 2.5 mg of dried CFS samples heated 30–500°C at a rate of 20°C/min, under a N_2_ atmosphere. Differential scanning calorimetry (DSC) was performed analyzing 10 mg of dry CFS in a Q2000 (TA Instrument, New Castle, DE, United States) at a rate of 10°C/min, under a nitrogen flow of 50 ml/min, at a 20–600°C range. For Gel permeation chromatography (GPC) of PE-PU-A copolymers, liquid CFS were extracted in a similar way that described above for GC-MS analysis, but the 2 ml chloroform:methanol (1:1, v/v) elution was evaporated to dryness at 25-30°C. The dried sample was resuspended in tetrahydrofuran (THF) at 15 mg/ml of solids, then filtrated through 0.45 μm Whatman filters and injected in a Waters 2695 Alliance Separation Module GPC (Milford, MA, United States) at 30°C in THF, using a universal column and a flow rate of 0.3 ml/min. All the analyses were performed at least in three replicates.

### Hi-C Proximity Ligation-Based Metagenomic Analysis

BP8 community cells cultured for 5 days in 50 ml of MM-PolyLack were harvested and washed three times with phosphate buffer. Cells were resuspended in 20 ml TBS buffer with 1% (v/v) formaldehyde (J. T. Baker) (crosslinker) and incubated 30 min with periodic mixing. The crosslinker was quenched with glycine (0.2 g) (Bio-Rad) for 20 min, thereafter cells were centrifuged, lyophilized and frozen at −20°C. For DNA extraction, cell pellets (100 μl of solid cellular material, equivalent to 10^9^ – 10^10^ cells) were resuspended in 500 μl of TBS buffer containing 1% (v/v) Triton X-100 and protease inhibitors (Press et al., 2017). DNA was digested with *Sau*3AI and MluCI and biotinylated with DNA Polymerase I Klenow fragment (New England Biolabs) followed by ligation reactions incubated for 4 h and then overnight at 70°C to reverse crosslinking. The Hi-C DNA library was constructed by using the HyperPrep Kit (KAPA Biosystems, Wilmington, MA, United States). A shotgun library was also prepared from DNA extracted from non-crosslinked cells using Nextera DNA Sample Preparation Kit (Illumina). The two libraries were paired-end sequenced using NextSeq 500 Illumina platform (Illumina, San Diego, CA, United States). *De novo* metagenome draft assemblies from the raw reads were made using the metaSPAdes assembler (Nurk et al., 2017). Hi-C reads were then aligned to the contigs obtained from the shotgun library using the Burrows-Wheeler Alignment tool (Li and Durbin, 2010) requiring exact read matching. The ProxiMeta algorithm was used to cluster the contigs of the draft metagenome assembly into individual genomes (Press et al., 2017). Additionally, we performed a community taxonomic profiling from shotgun reads using MetaPhlAn tool (Segata et al., 2012). Genome completeness, contamination, and other genomic characteristics were evaluated using CheckM pipeline (Parks et al., 2015). Phylogenetic analysis was performed using the single copy molecular markers, DNA gyrase subunit A and ribosomal proteins L3 and S5, selected from each deconvoluted genome and compared to homologous sequences from GenBank. Alignments were cured with Gblocks tool^1^ and WAG plus G evolutionary models were selected using Smart Model Selection tool (Lefort et al., 2017). Finally, phylogeny was inferred with the graphical interface of SeaView (Gouy et al., 2010) using the Maximum Likelihood method. To compare genetic relatedness, Average Nucleotide Identity (ANI) between the genomes and the closest phylogenetic neighbors was calculated (Yoon et al., 2017). Open reading frames were identified using MetaGeneMark (Zhu et al., 2010). KO assignments (KEGG Orthology) and KEGG pathways reconstruction were performed with GhostKOALA server and KEGG Mapper tool, respectively (Kanehisa et al., 2016). All the xenobiotic degradation pathways were manually curated to only report those pathways in which most of the enzymes were encoded in the BP8 metagenome.

## Results

### Growth and Interactions of BP8 Cells With PolyLack^®^

**FIGURE 1:**
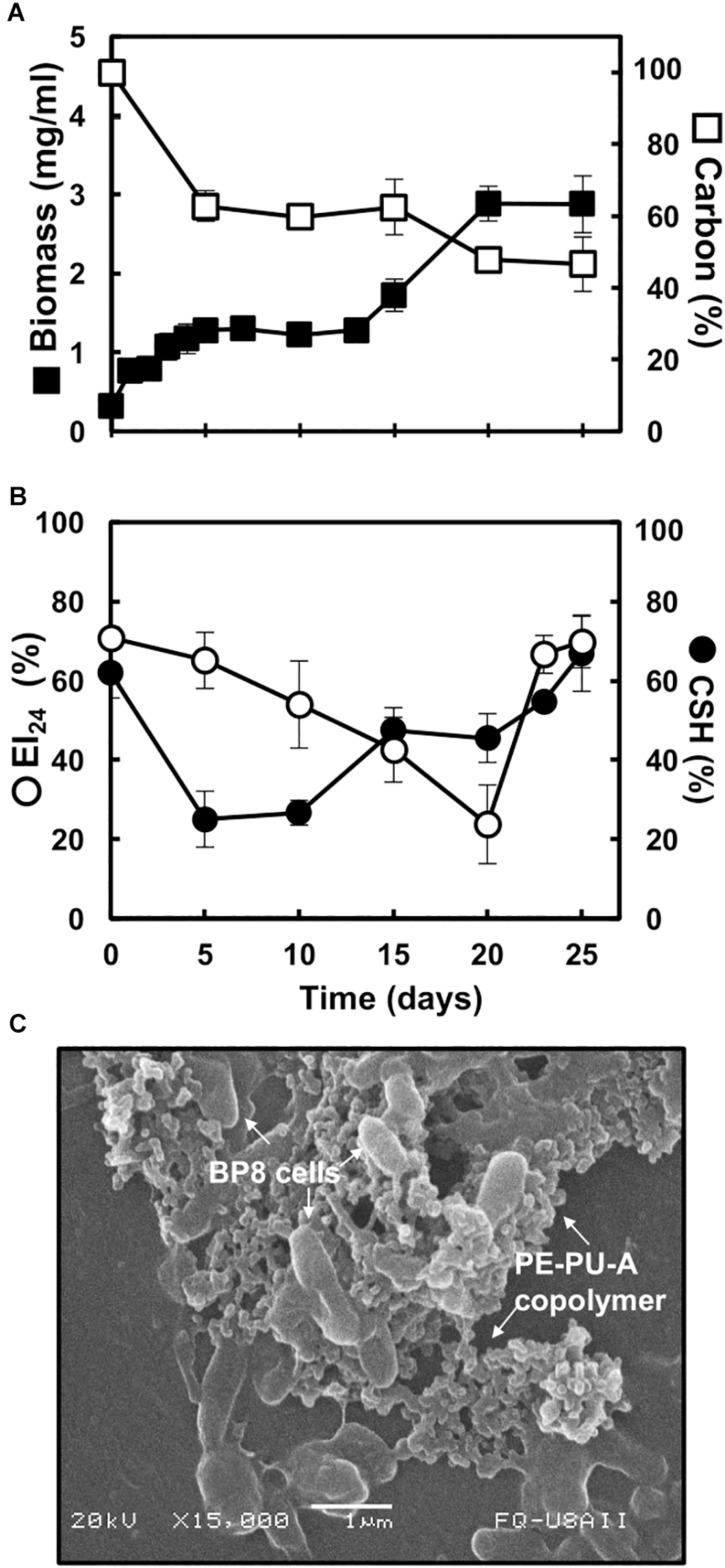
Characteristics of the BP8 community growing in MM-PolyLack. **(A)** Growth and carbon consumption, **(B)** emulsification index (EI_24_) and cell surface hydrophobicity (CSH) at different cultivation times. **(C)** SEM micrograph of BP8 cells attached to the PE-PU-A copolymer at 10 days of cultivation. Bars represent standard deviation. *n* = 3.

The BP8 community cultivated in MM-PolyLack for 25 days exhibited a biphasic growth with a first phase, from 0 to 13 days, presenting a growth rate (2–4 days) of 0.008 h^–1^ and a second phase, from 13 to 25 days, with a growth rate (13–20 days) of 0.005 h^–1^. Biomass increased from 0.32 to 2.9 mg/ml and consumed 50.3% of the carbon from the medium at 25 days (Figure 1A). EI_24_ initial value was 70%, it decreased to 24% at 20 days and increased again to 70%. CSH started at 62% and decreased to 25% at the first growth phase; thereafter it increased to 42% and remained constant until 20 days to increase to 67% at the end of the second phase (Figure 1B). SEM analysis at 10 days of cultivation revealed multiple-sized (0.5–1.5 μm) rod-shaped cells aggregated and attached to copolymer particles (Figure 1C).

### Chemical and Physical Changes in PolyLack^®^ Components Generated by the BP8 Community

**FIGURE 2:**
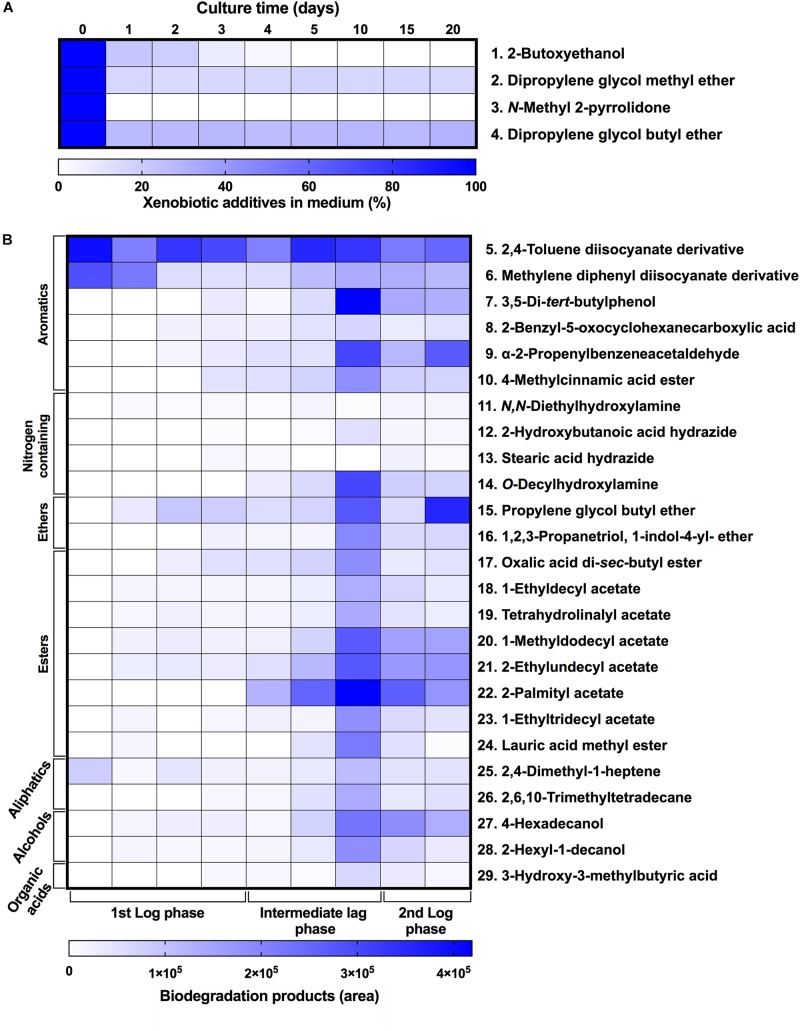
Xenobiotic additives consumed **(A)** and PE-PU-A biodegradation products generated **(B)** by the BP8 community. Cell-free supernatants were extracted at different cultivation times with chloroform:methanol and analyzed by GC-MS. **(A)** Additives were quantified using standard curves for each compound and **(B)** biodegradation products by analyzing their areas in independent chromatograms. *n* = 3. Compounds with mass spectra similarity values over 700 were considered the same compounds of the Library hits. The numbers in the compounds correspond to signals in the chromatograms of Supplementary Figure S1.

**FIGURE 3:**
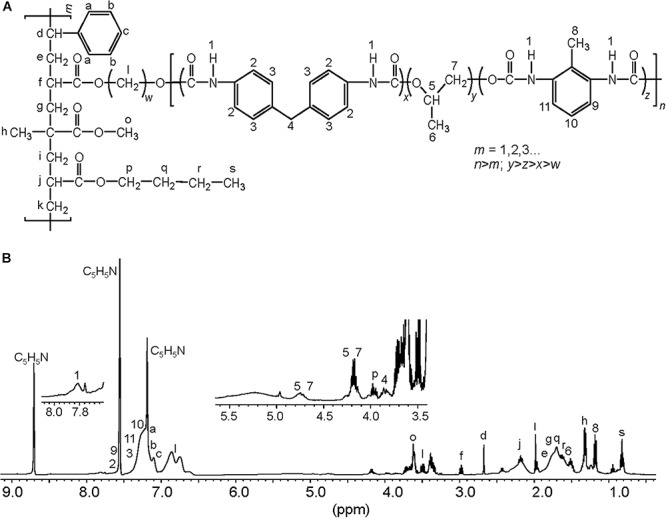
Proposed chemical structure for the PE-PU-A copolymer present in PolyLack^®^. **(A)** This structure was proposed based on the **(B)** ^1^H-NMR analysis of dried PolyLack^®^, the information included in the manufacturer technical manual (SayerLack. Poly Lack Aqua Brillante UB-0800), the GC-MS analysis (Figure 2), and the most frequent acrylates used in the synthesis of these types of copolymers (Pardini and Amalvy, 2008; Maurya et al., 2018). Synthesis of PE-PU-A copolymers starts by the polycondensation of polyols [polypropylene glycol (PPG)] (y moiety) and diisocyanates (TDI and MDI) (x and z moieties) followed by end capping with acrylates’ mixture (m moiety). From the most frequently used acrylates we selected methyl methacrylate, butyl acrylate, hydroxy acrylate and styrene as representatives in this structure. In the ^1^H-NMR spectrum, chemical shifts are provided in parts per million from SiMe_4_ as internal reference. Signal 1 is assigned to carbamate groups (NH-COO); signals a, b, c, 2, 3, 9-11 are assigned to the aromatic protons; signals 4 and 8 correspond to the protons of methylene (CH_2_) and methyl (CH_3_) groups in MDI and TDI, respectively; signals 5–7 correspond to PPG; signals l correspond to the hydroxyl proton (CH_2_-O) and methylene groups (CH_2_) in the chain of hydroxy-acrylate; signals f, j, o and p correspond to the acrylic groups (CH-COO, CH_2_-COO or CH_3_-COO), signals d (CH), e, g, i, k, q, r (CH_2_), h and s (CH_3_) are assigned to methylene and methyl groups in the acrylate mixtures.

**FIGURE 4:**
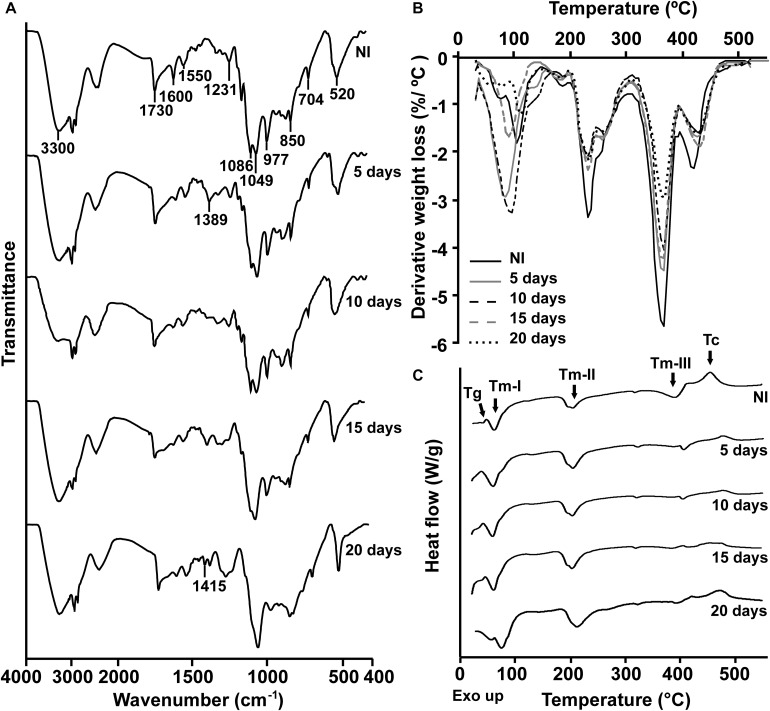
Physical and chemical analyses of the aromatic PE-PU-A copolymer after incubation with the BP8 community. **(A)** FTIR spectra. **(B)** DTG analysis. Thermal degradation stages correspond to the following functional groups: (I) Low molecular weight compounds, (II) Urethane, (III) Ester, (IV) Ether. **(C)** DSC analysis. Glass transition temperature (Tg) represents the relative amount of soft and hard segments; melting temperatures, Tm-I, Tm-II, and Tm-III are associated with hard domains, and crystallization temperature (Tc) represents heat-directed crystallization of copolymer chains. NI, non-inoculated.

To characterize the biodegradative activity of the BP8 community on the PolyLack^®^ components, we performed different analytical techniques. GC-MS analysis of the CFS revealed that BP8 metabolized the xenobiotic additives, NMP and IP at the first day of cultivation and 2-BE at the fourth day. DPGM and DPGB were metabolized 84 and 73%, respectively, at the first day and remained constant until the end of the experiment (Figure 2A and Supplementary Table S1). Since the PE-PU-A copolymer structure is unknown, we proposed a hypothetical structure (Figure 3), based on ^1^H-NMR, the manufacturer’s technical sheet and in the most frequently used chemicals for the synthesis of this copolymer (Pardini and Amalvy, 2008; Gite et al., 2010; Maurya et al., 2018). Since the first day of cultivation, complex and diverse chemical compounds such as aromatics, nitrogen-containing, ethers, esters, aliphatics, alcohols and organic acids, derived from the copolymer breakdown were observed. During the first 3 days (log phase) the degradation products were low abundant, at 10 days (intermediate lag phase) accumulation occurred, and during the second log phase their abundance decreased. Notably, isocyanates [2,4-toluene diisocyanate (TDI) and methylene diphenyl diisocyanate (MDI)] derivatives were aromatic amines observed maximal at the beginning and diminished throughout the cultivation period (Figure 2B, Supplementary Figure S1, and Supplementary Table S2), suggesting that metabolization of the urethane groups is being achieved. FTIR of dried CFS revealed changes in PE-PU-A functional groups. The signal intensity of the C=O stretch from urethane and acrylate carbonyl groups (1730 cm^–1^) increased at 5 days and lately decreased, suggesting hydrolysis and subsequent catabolism of urethanes and acrylates. The signal for aromatic groups C=C stretch (1600 cm^–1^) considerably decreased at 20 days, while the signal for aromatic C-C stretch (1380 cm^–1^) showed variable intensities at different days, and a new C-C signal for aromatics (1415 cm^–1^) appeared at 20 days, indicating the cleavage of the aromatic rings. The urethane N-H bending plus C-N stretch signal (1550 cm^–1^) slightly decreased at 15 days and increased at the end of the cultivation time, whereas urethane C-N stretching band (1231 cm^–1^) significantly increased, indicating urethane attack. Signals associated with urethane C-O-C stretch (1086 cm^–1^, 1049 cm^–1^) and C-O-C symmetric stretch (977 cm^–1^) decreased during the cultivation period, indicating microbial activity on the ether groups. The signal for the acrylate’s vinyl group C=CH_2_ out of plane (850 cm^–1^) decreased at 20 days, indicating the cleavage of the acrylate component. Also, the aliphatic chain signals (704 and 520 cm^–1^) decreased during the cultivation period (Figure 4A). DTG thermograms exhibited four stages of thermal decomposition corresponding to the functional groups of the copolymer. Stages II and IV, for urethane and ether groups respectively, reduced their masses at early cultivation times, while stage III, for esters, steadily kept reducing its mass during the whole experimental period. Interestingly, stage I, which accounts for low molecular weight compounds, in this case biodegradation products, showed a fluctuating behavior that increased at 10 days, and decreased afterward (Figure 4B). DSC analysis of the copolymer showed multiple thermal transitions revealing complex microstructures: the glass transition temperature (Tg: 50.2°C) reflects the proportion of soft and hard segments; the three melting temperatures (Tm-I: 70°C, Tm-II: 210.6°C, Tm-III: 398.1°C) are associated with the hard segments of the polymer and the crystallization temperature (Tc: 459.6°C) is attributed to heat-directed crystallization of copolymer chains (Cipriani et al., 2013; Sultan et al., 2014) (Figure 4C). BP8 biodegradative activity caused Tg decrease (46.2°C), changes in Tms, and strong decrease in Tc area, indicating that BP8 disrupts both, the soft and the hard segments (associated with urethane groups) (Figure 4C and Supplementary Table S3). GPC analysis showed that the number-average molecular weight (*Mn*) of the copolymer steadily decreased 35.6% up to the end of the culture time, meanwhile the weight-average molecular weight (*Mw)* increased about 10%, from 0 to 15 days of cultivation, and then decreased 26% from 15 to 25 days. The Polydispersity index (PDI) increased over 2, at 25 days of cultivation with BP8. Analysis of Molecular weight distribution (MWD) of the degraded samples showed shifts toward higher molecular weights than control up to 20 days of analysis. However, at 25 days, a strong shift to lower molecular weights was observed (Table 1 and Supplementary Figure S2). Abiotic controls of different cultivation times were evaluated and no changes were observed (Table 1). All these results indicate that the degradative activity of the BP8 community generated changes in the soft and hard segments of the copolymer microstructure resulting from the attack to the different functional groups, including the more recalcitrant ether and urethane groups.

**TABLE 1:** Molecular weight and polydispersity index of the PE-PU-A copolymer during cultivation with the BP8 community.

**Culture time**	***Mn***	***Mw***	**PDI**
**(days)**	**(g/mol)**	**(g/mol)**	
Non-inoculated	101,8968,098	208,4907,600	2.00.3
5	96,798712	210,83726,098	2.20.1
10	89,2585,825	223,58118,736	2.50.3
15	82,5771,168	229,37226,416	2.70.3
20	73,4069,225	187,92550,737	2.40.3
25	65,609990	169,9255,080	2.60.2

*Number-average molecular weight (*Mn*), weight-average molecular weight (*Mw*) and polydispersity index (PDI) were calculated by Gel permeation chromatography with monodisperse polystyrene calibration standards and using THF as eluent. *n* = 3.*

### Community Structure and Metagenomic Deconvolution of the BP8 Community

**FIGURE 5:**
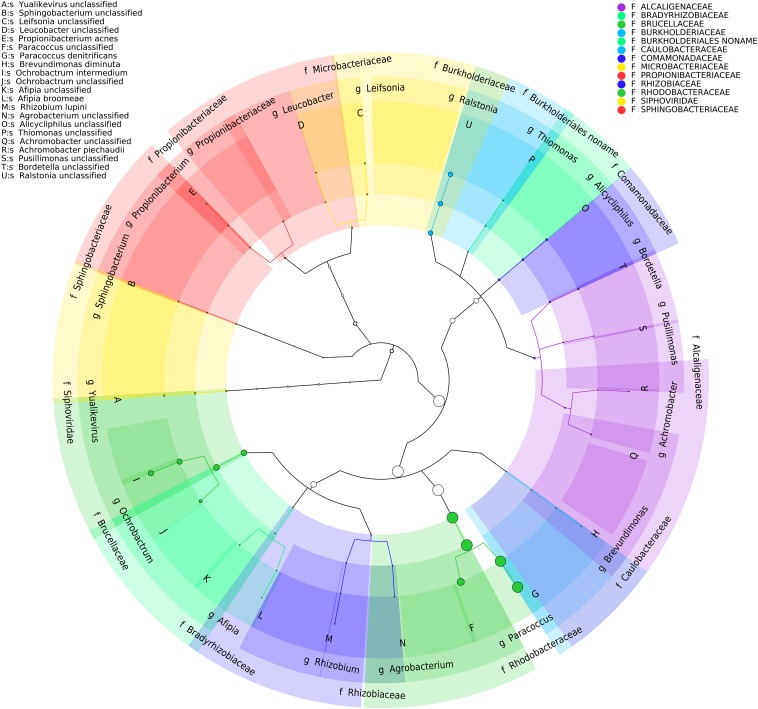
Taxonomic cladogram of BP8 community microbial diversity profiled with MetaPhlAn. Circles size is proportional to the taxon relative abundance. The most abundant taxa were *Paracoccus* genus (83%) and *Ochrobactrum* genus (8.7%). Families are color-labeled and predicted species diversity is indicated by capital letters (Asnicar et al., 2015).

**FIGURE 6:**
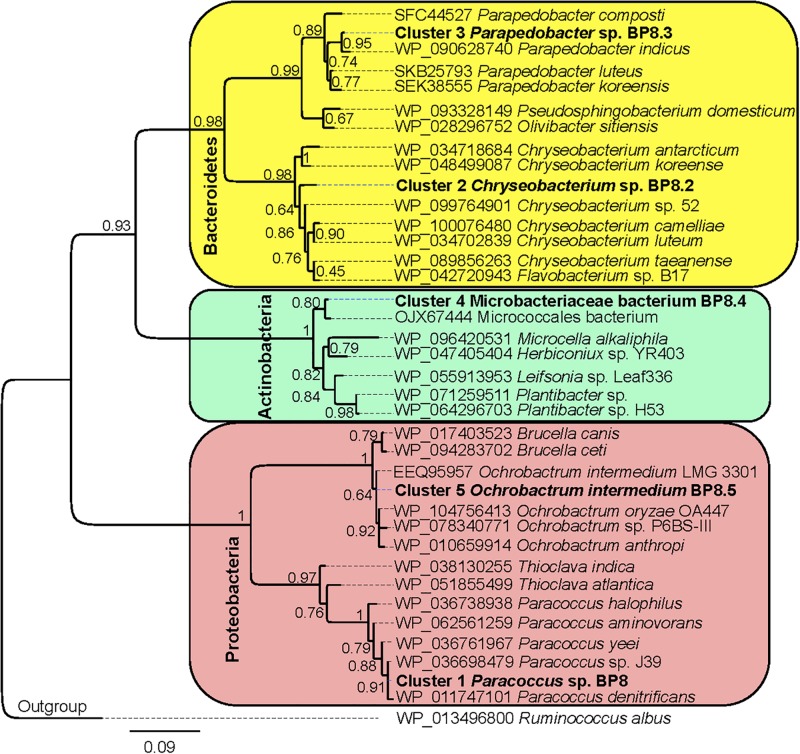
Maximum likelihood phylogeny for taxonomic delimitation of the deconvoluted genomes from the BP8 metagenome. The analysis was performed with three phylogenetic markers: ribosomal protein L3, ribosomal protein S5 and DNA gyrase A subunit, which generated similar results. The analysis for ribosomal protein L3 is presented. Branch support values are indicated in the corresponding nodes. Bar indicates the number of expected substitutions per site under the WAG + G model. A sequence of *Ruminococcus albus* (Firmicutes) was used as outgroup. Key genome clusters are highlighted in bold and different Phyla are indicated at the left. Sequences for L3 ribosomal proteins of the deconvoluted genomes are accessible in the NCBI GenBank under accession numbers RQP07704.1, RQP15098.1, RQP16503.1, RQP08603.1, and RQP16393.1 for clusters 1–5, respectively.

Analysis of the BP8 community taxonomic profile with MetaPhlAn, by using 17,282,414 reads, detected five bacterial orders (abundance), *Rhodobacterales* (83%), *Rhizobiales* (8.9%), *Burkholderiales* (6.8%), *Actinomycetales* (0.83%), *Sphingobacteriales* (0.08%), and one viral order *Caudovirales* (0.33%). Bacteria included 16 genera, being the most abundant *Paracoccus* (83%) and *Ochrobactrum* (8.7%) (Figure 5). The shotgun Illumina library was used to create a draft *de novo* metagenome assembly. After parsing contigs lesser to 1000 bp, this assembly had 5339 contigs with 21,228,807 bp in size. Subsequently, we mapped the Hi-C reads to the draft shotgun assembly generating 3,072 contigs with a total length of 17,618,521 bp. The alignment of Hi-C reads to this assembly allowed the deconvolution of five genome clusters, three near complete drafts (completeness >95%), and two substantially complete drafts (completeness 89 and 71%) (Parks et al., 2015) (Table 2). The phylogenetic analysis showed well-supported clades within *Paracoccus*, *Chryseobacterium*, *Parapedobacter*, a member of the *Microbacteriaceae* family, and *Ochrobactrum intermedium* (Figure 6). The deconvoluted genomes of *Paracoccus* sp. BP8 and *O. intermedium* BP8.5 showed low novelty scores and high ANI values compared to their closest phylogenetic relatives. In contrast, *Chryseobacterium* sp. BP8.2, *Parapedobacter* sp. BP8.3 and the *Microbacteriaceae* bacterium BP8.4 showed high novelty scores and low ANI values (<95%) indicating they are new species. GC content and genomes’ sizes were similar to the closest relatives except for the *O. intermedium* BP8.5 genome size, probably because of the low genome completeness (Table 2 and Supplementary Table S4).

**TABLE 2:** General features of the deconvoluted genomes from the BP8 metagenome.

**Cluster ID**	**Genome Size (bp)**	**Num Contigs**	**Contig N50**	**^a^Completeness (%)**	**^b^Relative Abundance (%)**	**Novelty Score (%)**	**GC (%)**	**Identification**	**Genes assigned**	**Proteins assigned**
1	4,275,656	282	51,004	89.4	57.7	1.6	67.8	*Paracoccus* sp. BP8	4,225	4,073
2	2,157,639	388	7,081	95.6	3.7	98.7	47.3	*Chryseobacterium* sp. BP8.2	2,253	2,185
3	5,478,545	1098	6,493	95.5	12.5	99.2	48.1	*Parapedobacter* sp. BP8.3	5,310	5,173
4	2,790,120	158	39,967	97.7	3.6	94.0	71.3	**^c^***Microbacteriaceae* bacterium BP8.4	2,850	2,705
5	2,916,513	1146	2,823	71.0	22.5	2.5	58.4	*Ochrobactrum intermedium* BP8.5	3,472	3,162

*^*a*^Completeness was calculated based on 40 single copy gene markers (Parks et al., 2015). All the genomes’ drafts have at least 18 tRNAs and, except for cluster 5, at least one rDNA gene copy. ^*b*^Relative abundance was normalized according to the reads distribution along the deconvoluted taxon. **^*c*^** For *Microbacteriaceae* bacterium BP8.4 no further classification was possible even that nine single-copy markers were analyzed.*

### Analysis of the Xenobiotic Metabolism Encoded in the BP8 Metagenome

In all the genomes, except in *O. intermedium* BP8.5, the genes and proteins assigned were in the range reported for the phylogenetically related members (Table 2 and Supplementary Table S4). Reconstruction of the metabolic pathways encoded in the BP8 metagenome was performed with 18,386 ORFs from which 8,637 were annotated into KEGG Orthology groups and the rest was not assigned to any orthologous functional category. Analysis of the BP8 xenobiotic metabolism identified 215 sequences encoding 59 unique proteins participating in pathways for benzoate (ko00362), fluorobenzoate (ko00364), aminobenzoate (ko00627), chlorocyclohexane and chlorobenzene (ko00361), and *n*-alkanes (ko00071) degradation. The most relevant enzymes are listed in Table 3. The genes for benzoate metabolism include all the enzymes for benzoate and 4-methoxybenzoate activation as well as 4-methoxybenzoate monooxygenase, an O-demethylating enzyme that transforms methoxybenzoate to hydroxybenzoate, and for their subsequent transformation to β-ketoadipate (first 18 EC numbers in Table 3). Two genes encoding carboxymethylene butanolidase that cleaves the ring of cyclic ester dienelactone to produce maleylacetate, acting on the fluorobenzoate and chlorocyclohexane and chlorobenzene metabolisms, were identified. Genes encoding enzymes for the aminobenzoate pathway, such as 4-hydroxybenzoate decarboxylase that participates in the transformation of phenol into hydroxybenzoate, amidase that transforms benzamide into benzoate, and benzoyl phosphate phospohydrolase that converts benzoyl phosphate into benzoate, were identified. All the genes encoding enzymes needed for chlorocyclohexane and chlorobenzene degradation, the specific 2,4-dichlorophenol 6-monooxygenase, the enzymes that transform 4-chlorophenol to *cis*-acetylacrylate (EC 1.13.11.1, EC 5.5.1.1, and EC 3.1.1.45), and the 2-haloacid dehalogenase, which eliminates halogens from alkanes, were found. Likewise, genes encoding enzymes for *n*-alkanes degradation (Table 3 Alkanes metabolism), as well as allt he enzymes for beta-oxidation were also detected.

**TABLE 3:** Distribution of genes encoding relevant proteins involved in xenobiotics degradation in the BP8 metagenome.

**Activity**	**K Number**	**EC**	**Name**	***Paracoccus* sp. BP8**	***Chryseobacterium* sp. BP8.2**	***Parapedobacte*r sp. BP8.3**	**Microbacteriaceae bacterium BP8.4**	***O. intermedium* BP8.5**
**Benzoate and related compounds metabolism**								
Benzoate/toluate 1,2- dioxygenase	K05549 K05550 K05784	1.14.12.10 1.18.1.-	*benA-xylX benB-xylY benC-xylZ*	1 1 1	– – –	– – –	– – –	– – –
Dihydroxyclohexadiene carboxylate dehydrogenase	K05783	1.3.1.25	*benD-xylL*	1	–	–	–	–
*p*-Hydroxybenzoate 3-monooxygenase	K00481	1.14.13.2	*pobA*	1	–	–	–	1
Catechol 1,2-dioxygenase	K03381	1.13.11.1	*catA*	1	–	–	–	–
Catechol 2,3-dioxygenase	K07104	1.13.11.2	*catE*	1	–	–	1	1
Protocatechuate 3,4-dioxygenase	K00448 K00449	1.13.11.3	*pcaG-pcaH*	1 1	– –	– –	– –	1 1
Muconate cycloisomerase	K01856	5.5.1.1	*catB*	1	–	–	1	–
3-Carboxy-*cis,cis*-muconate cycloisomerase	K01857	5.5.1.2	*pcaB*	1	–	–	–	1
Muconolactone isomerase	K01856	5.3.3.4	*catC*	1	–	–	–	–
4-Carboxymuconolactone decarboxylase	K01607	4.1.1.44	*pcaC*	2	–	–	–	1
Enol-lactone hydrolase	K01055	3.1.1.24	*pcaD*	3	–	–	–	1
β-ketoadipate:succyinyl- CoA transferase,	K01031 K01032	2.8.3.6	*pcaI-pcaJ*	1 1	– –	– –	– –	2 1
β-ketoadipyl-CoA thiolase	K00632	2.3.1.16	*pcaF*	–	1	1	1	1
2-Oxopent-4-enoate hydratase (benzoate)	K02554	4.2.1.80	*mhpD*	–	–	–	1	–
4-Hydroxy 2-oxovalerate aldolase	K01666	4.1.3.39	*mhpE*	–	–	–	2	–
Acetaldehyde dehydrogenase	K04073	1.2.1.10	*mhpF*	–	–	–	2	–
4-Methoxybenzoate monooxygenase (*O*-demethylating)	K22553	1.14.99.15	CYP199A2	1	–	–	–	–
Carboxymethylene butanolidase	K01061	3.1.1.45	*clcD*	–	–	–	1	1
4-Hydroxybenzoate decarboxylase	K03186	4.1.1.61	*ubiX*	2	–	1	–	1
Amidase	K01426	3.5.1.4	*amiE*	1	–	–	1	1
Benzoyl phosphate phosphohydrolase	K01512	3.6.1.7	*acyP*	–	–	1	1	–
2,4-Dichlorophenol 6-monooxygenase	K10676	1.14.13.20	*tfdB*	1	–	–	–	–
2-Haloacid dehalogenase	K01560	3.8.1.2		2	–	–	–	1
**Alkanes metabolism**								
Alkane 1-monooxygenase	K00496	1.14.15.3	*alkB1_B2 alkM*	2	–	–	–	–
Ferredoxin NAD^+^ reductase component	K00529	1.18.1.3	*hcaD*	2	–	–	–	1
Unspecific monooxygenase	K00493	1.14.14.1		2	–	–	–	1
Long-chain-alkane monooxygenase	K20938	1.14.14.28	*LadA*	–	–	–	1	–
Alcohol dehydrogenase propanol preferring	K13953	1.1.1.1	*adhP*	2	–	1	–	1
Aldehyde dehydrogenase (NAD^+^ dependent)	K00128	1.2.1.3	ALDH	6	2	–	–	3
Aldehyde dehydrogenase (NADP dependent)	K14519	1.2.1.4	*aldH*	–	1	–	–	–
Lipocalin family protein	K03098	–	*Blc*	–	1	1	–	1
Long chain fatty acid transport protein	K06076	–	*fadL*	1	–	–	–	–
**N-methylpyrrolidone metabolism**								
*N*-methylhydantoin amidohydrolase	K01473 K01474	3.5.2.14	*nmpA nmpB*	5 5	– –	– –	– 1	1 –
Aminoacid oxidase	–	–	*nmpC*	3	–	–	2	1
Succinate-semialdehyde dehydrogenase	K00135	1.2.1.16	*nmpF*	7	–	1	2	1
**Isopropanol metabolism**								
^a^Alcohol dehydrogenase propanol preferring	K13953	1.1.1.-	*adh1*	1	–	–	–	–
Alcohol dehydrogenase ^b^Aldehyde dehydrogenase	K18369 K00138 K10854	1.1.1.- 1.2.1.-	*adh2 adh3 acxB*	2 1 1	– – –	– – –	– 1 –	– – –
Acetone carboxylase	K10855 K10856	6.4.1.6	*acxA acxC*	1 1	– –	– –	– –	1 –
3-Oxoacid-CoA transferase	K01028 K01029	2.8.3.5		1 1	1 1	1 1	1 1	– –
Acetoacetate-CoA ligase	K01907	6.2.1.16	*acsA*	–	–	–	–	1
Acetyl-CoA C-acetyltransferase	K00626	2.3.1.9	*atoB*	10	1	1	3	5
**Glycol ethers and polypropylene glycols metabolism**								
^c^Alcohol dehydrogenase,	–	1.1.1.-	*pegdh*	3	–	–	–	3
^d^Aldehyde dehydrogenase	–	1.2.1.3	*pegC*	3	–	–	1	–
Glycolate oxidase	K00104 K11472 K11473	1.1.3.15	*glcD glcE glcF*	1 1 1	– – –	– – –	– – 1	2 1 1
Superoxide dismutase	K04564 K04565	1.15.1.1	SOD2 SOD1	– 1	1 1	3 –	1 –	1 –
Dye decoloring peroxidase	K15733	1.11.1.19	*DyP*	1	–	–	1	–
Glutathione S-transferase	K00799	2.5.1.18	*gst*	11	–	–	–	8
Acyl Co-A synthetase	K01897	6.2.1.3	*ACSL*	2	3	2	2	1
*S*-(hydroxymethyl) glutathione dehydrogenase	K00121	1.1.1.284	*frmA*	3	–	–	–	2
*S*-formylglutathione hydrolase	K01070	3.1.2.12	*fghA*	1	–	–	–	1

*^*a*^*Adh1* was identified by BLAST analysis using the *adh1* sequence (Acc. num. BAD03962.1) reported for *Gordonia* sp. TY-5 (Kotani et al., 2003) as query (Query cover >99%; *E*-value = 4E-42; Identity 34%). The gene accession number in the BP8 metagenome is RQP06405.1. ^*b*^*Adh3* genes were identified by BLAST analysis using the *adh3* sequence (Acc. num. BAD03965.1) reported for *Gordonia* sp. TY-5 (Kotani et al., 2003) as query (Query cover = 97%; *E*-value = 1E-97; Identity = 38.8%). The gene accession numbers in the BP8 metagenome are RQP06404.1 and RQP13157.1. These genes were classified as aldehyde dehydrogenases by KEGG, similarly as described in Kotani et al. (2003). ^*c*^*Pegdh* genes were identified by BLAST analysis using the polyethylene glycol dehydrogenase sequence (*pegdh*) from *Sphingopyxis terrae* (Acc. num. BAB61732) (Ohta et al., 2006) as query (Query cover = 97%; *E*-value = 2.0E-122; Identity = 38.5%). The gene accession numbers in the BP8 metagenome are RQP05609.1, RQP06903.1, RQP07092.1, RQP19606.1, RQP18819.1, RQP20974.1. ^*d*^*Pegc* genes were identified by BLAST using polyethylene glycol aldehyde dehydrogenase sequence from *Sphingopyxis macrogoltabida* (Acc. num. BAF98449.1) (Tani et al., 2007) as query (Query cover = 98%; *E*-value = 1.0E-80; Identity = 38%; Similarity = 56%). The gene accession numbers in the BP8 metagenome are RQP06197.1, RQP04172.1, RQP06015.1, RQP13157.1. Only RQP06197.1 was identified as K00128 by KEGG.*

### BP8 Community Phenotypic Potential to Biodegrade the Xenobiotic Additives of PolyLack^®^

#### NMP Degradation

Genes encoding putative proteins for NMP degradation, with significant similarity (>40%) to the enzymes of *Alicycliphilus denitrificans* BQ1 (Solís-González et al., 2018) were identified in several BP8 genomes (Table 3). However, only in *Paracoccus* sp. BP8 a gene cluster (RQP05666.1–RQP05671.1) comparable to the BQ1 *nmp* cluster was identified.

#### Isopropanol Degradation

**FIGURE 7:**
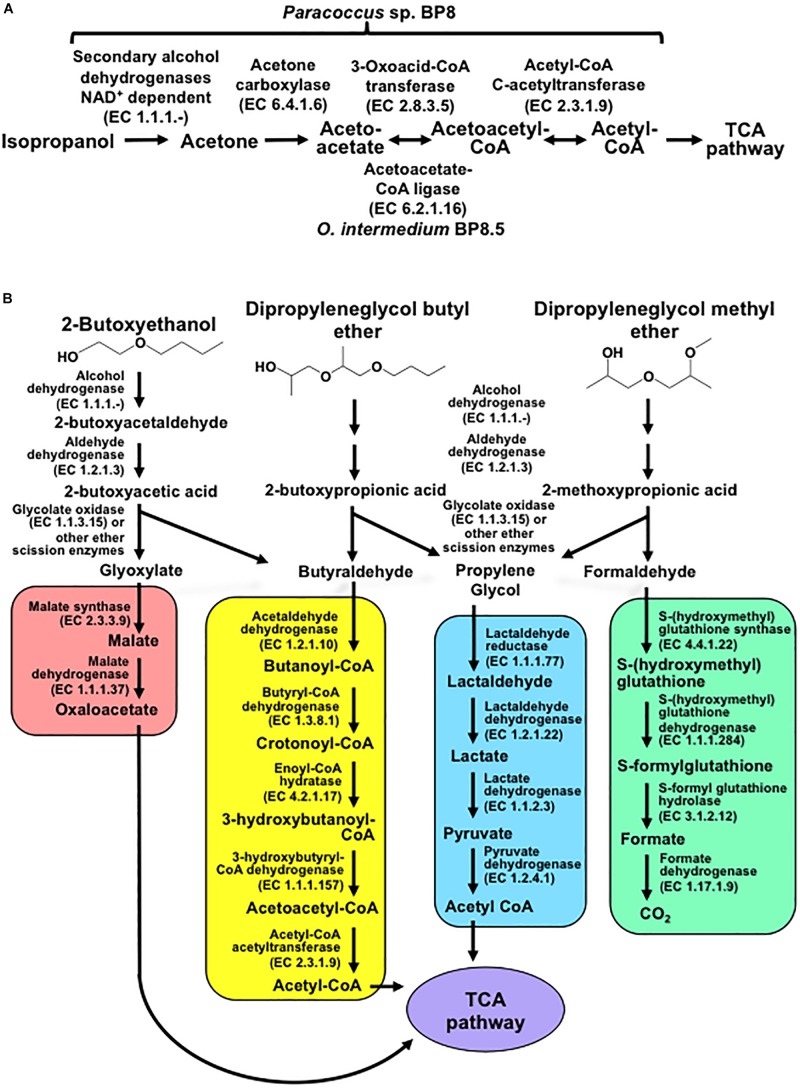
Potential degradation pathways for isopropanol **(A)** and glycol ethers **(B)** encoded in the BP8 metagenome. **(A)** *Paracoccus* sp. BP8 genome encodes ADH enzymes that can oxidize IP to acetone, but genes encoding enzymes for the oxidative metabolism of acetone were not found. Instead, genes encoding the reductive transformation of acetone to acetyl CoA, acetone carboxylase, 3-oxoacid-CoA transferase and acetyl-CoA C-acetyltransferase, were identified. In *O. intermedium* BP8.5 genome, acetoacetate-CoA ligase, which performs the same reaction that 3-oxoacid-CoA transferase, is encoded. All the enzymes for the TCA pathway are encoded in the BP8 metagenome (see Table 3). **(B)** Subsequent oxidations of glycol ethers’ hydroxy terminal groups by PEG-DH and PEG-ALDH will produce 2-butoxyacetic acid from 2-BE, 2-butoxypropionic acid from DPGB and 2-methoxypropionic acid from DPGM. Subsequent cleavage of carboxylates’ ether bonds by ether scission enzymes such as glycolate oxidase, dye decoloring peroxidase, 4-methoxybenzoate monooxygenase and/or unspecific monooxygenase, would generate the metabolizable intermediaries glyoxylate, butyraldehyde, propylene glycol and formaldehyde. Glyoxylate would be funneled to the glyoxylate metabolism (red rectangle), butyraldehyde to the butanoate metabolism (yellow rectangle), propylene glycol to the pyruvate metabolism (blue rectangle), and formaldehyde to the formate metabolism (green rectangle). Pathways for glyoxylate, butanoate, pyruvate and formate metabolisms as well as the TCA pathway (purple ellipse) were fully reconstructed from the BP8 metagenome based on KEGG annotated genes, using KEGG Mapper.

Genes encoding proteins with significant similarity to NAD^+^-dependent secondary alcohol dehydrogenase (ADH) with capability to oxidize IP to acetone were identified in the BP8 metagenome (Kotani et al., 2003), but not the genes encoding the enzymes for the oxidative transformation of acetone. However, the three genes encoding acetone carboxylase that transforms acetone into acetoacetate were identified. Similarly, the genes encoding 3-oxoacid-CoA transferase, in *Paracoccus* sp. BP8, and acetoacetate-CoA ligase, in *O. intermedium* BP8.5, that convert, both of them, acetoacetate into acetoacetyl-CoA, were observed. Besides, genes for acetyl-CoA C-acetyltransferase, which transforms acetoacetyl CoA to acetyl CoA that enters the TCA pathway, were also found in the BP8 metagenome (Figure 7A and Table 3).

#### Glycol Ethers Degradation

In the BP8 metagenome, homologous genes to polyethylene glycol (PEG)-degrading ADHs and aldehyde dehydrogenases (ALDHs) (Ohta et al., 2006; Tani et al., 2007), and diverse enzymes that could attack the ether bonds, such as glycolate oxidase (RQP04511.1, RQP04512.1, RQP04513.1, RQP11464.1, RQP19624.1, RQP19625.1, RQP16322.1, RQP16256.1), dye decoloring peroxidase (RQP04907.1, RQP09154.1) and superoxide dismutase (RQP04715.1, RQP13424.1, RQP09887.1, RQP11889.1, RQP18047.1, RQP18034.1, RQP09190.1, RQP20377.1), as well as genes encoding enzymes involved in glutathione metabolism, which have been proposed to participate in PEG metabolism (Somyoonsap et al., 2008) were identified (Figure 7B and Table 3).

### BP8 Community Phenotypic Potential to Biodegrade Polyurethane

Genes encoding PU-esterases verified for PU degradation (Nomura et al., 1998; Stern and Howard, 2000; Ufarté et al., 2017) and confirmed carbamate-hydrolyzing enzymes, i.e., arylamidase A (Zhang et al., 2012), amidase (Yun et al., 2016), urethanase (Liu et al., 2016), and carbaryl hydrolase (Hashimoto et al., 2006), were searched by standalone BLASTP analyses. Six and five sequences with similarity to PU-esterases and carbamate hydrolases were retrieved from the BP8 metagenome, respectively (Table 4). We also identified genes encoding ureases (EC 3.5.1.5), suggested to act on PU degradation (Yang et al., 2015), in *Parapedobacter* sp. BP8 (RQP19536.1, RQP19537.1 RQP19538.1) and *O. intermedium* BP8.5 (RQP17756.1, RQP17448.1, RQP17449.1, RQP17450.1) genomes.

**TABLE 4:** Esterases and carbamate hydrolyzing enzymes encoded in the BP8 metagenome.

**Enzyme Query Organism (Accession number)**	**E.C. num.**	**Amino acids in the query**	**Hit in the BP8 metagenome**	**E value/ ^a^Identity/Similarity**	**Amino acids in the hit**	**References**
Polyurethane esterase *Delftia acidovorans* (BAA76305)	3.1.1.6	548	*Parapedobacter* sp. BP8.3 (RQP17780.1)	1.0E−07/32%/48%	640	Nomura et al., 1998
Polyurethanase esterase A *Pseudomonas chlororaphis* (AAD22743)	3.1.1.-	617	*Paracoccus* sp. BP8 (RQP07762.1)	4.0E−16/34%/50%	783	Stern and Howard, 2000
			*Paracoccus* sp. BP8 (RQP06646.1)	2.0E−14/33%/46%	980	
			*Paracoccus* sp. BP8 (RQP04598.1)	7.0E−12/30%/43%	854	
			*Paracoccus* sp. BP8 (RQP06839.1)	5.0E−12/32%/43%	612	
Esterase CE_Ubrb uncultured bacterium (SIP63154)	3.1.1.-	295	*Microbacteriaceae* bacterium BP8.4 (RQP12977.1)	8.0E−05/35%/48%	309	Ufarté et al., 2017
Arylamidase A *Paracoccus huijuniae* (AEX92978)	3.4.11.2	465	*Paracoccus* sp. BP8 (RQP04489.1)	6.0E−25/41%/52%	471	Zhang et al., 2012
Amidase *Ochrobactrum* sp. TCC-2 (ANB41810)	3.5.1.4	474	*Microbacteriaceae* bacterium BP8.4 (RQP11486.1) *O. intermedium* BP8.5 (RQP19215.1)	2.0E−47/35%/49% 6.0E−24/39%/50%	475 326	Yun et al., 2016
Urethanase *Lysinibacillus fusiformis* (KU353448)	3.5.1.4	472	*Microbacteriaceae* bacterium BP8.4 (RQP12064.1)	1.0E−60/32%/48%	499	Liu et al., 2016
Carbaryl hydrolase *cahA Arthrobacter* sp. RC100 (BAC15598)	3.6.3.5	506	*Paracoccus* sp. BP8 (RQP06118.1)	1.0E−15/35%/48%	326	Hashimoto et al., 2006

## Discussion

### The BP8 Microbial Community Displays a Diauxic Growth Phase That Seems Not to Be Based on the Utilization of Different Carbon Sources

To elucidate the mechanisms that landfill microbial communities perform to degrade the recalcitrant PE-PU plastic, we studied the degradative activity of the selected BP8 microbial community because of its capability to grow in PolyLack^®^. PolyLack^®^ is a WPUD that contains a proprietary PE-PU-A copolymer and several xenobiotic additives (NMP, IP, 2-BE, DPGB and DPGM). Chemical and physical analyses demonstrated that BP8 consumes the additives and breaks the copolymer. Hi-C based metagenomic analysis allowed us to unveil the phenotypic potential to degrade PU and xenobiotics of five deconvoluted genomes from the community. The diauxic growth of BP8 observed during 25 days of cultivation in MM-PolyLack suggested that two different metabolic processes were involved in degrading the components of the WPUD. We hypothesized that the additives were consumed during the first phase whereas the copolymer was broken during the second one. However, the biomass increment and the carbon decrease observed in the first growth phase (Figure 1A) resulted not only from additive consumption, but also from the copolymer breakdown (Figures 2, 4, Table 1, Supplementary Figure S1, and Supplementary Tables S1, S2). The simultaneous degradation of additives and copolymer observed in this analysis do not account for the diauxic behavior of the community. Interestingly, based on the slight *Mw* increase and the continuous *Mn* decrease of the copolymer observed during the first 15 days of cultivation, clearly observed in the MWD analysis, we suggest that BP8 community might preferentially attack low molecular weight fractions. Thus, the larger molecules would remain in the culture media and would be cleaved after 15 days of cultivation as evidenced by the continuous decrease in *Mn* and the steep decrease on *Mw* observed from 15 to 25 days (Table 1 and Supplementary Figure S2). Changes in these parameters resulting from polymer biodegradation have been reported for polyethylene (Albertsson et al., 1998) and polystyrene (Yang et al., 2018). Therefore, it seems that an enhanced PU-degrading community, able to attack more complex polymers could be selected after 15 days of cultivation, coincidental with the second exponential growth phase. Further studies to test this possibility are needed.

### The BP8 Microbial Community Exhibits Variable Cell-Surface Interactions That Could Be Involved in the Biodegradation Process

PolyLack^®^ additives and copolymer, as well as the degradation products generated by BP8 activity, have effects on the emulsifying capacity of the culture medium, consequently in cell-substrate interactions that have impact on the biodegradation process. PolyLack^®^ has intrinsic emulsifying properties provided by the additives and PE-PU-A copolymers (Honarkar, 2018), which determine the initial EI_24_ of the culture medium (70%) (Figure 1B). The initial and almost complete additives degradation during the first 5 days of cultivation accounts for a 5% decrement in the EI_24_ (Figure 2A and Supplementary Table S1). The larger loss of the emulsifier capacity observed from 5 to 20 days must be the result of hydrophilic moieties cleavage from the copolymer. The further increase observed in this parameter might be generated by BP8 biosurfactant production. Surfactants enhance substrate bioaccesibility and modify cell hydrophobicity improving biodegradation (Tzintzun-Camacho et al., 2012). CSH reflects the capability of BP8 cells to attach the PE-PU-A hydrophobic moieties and other hydrophobic components of the culture medium, favoring cell-substrate interactions that enhance biodegradation. The variability in CSH behavior during the cultivation period is consequence of modifications on cell envelopes composition and properties induced by changes in the culture medium. The changes in CSH and EI_24_ observed in this experiments reveal the complex cell-substrate interactions involved in promoting BP8 accessibility to PolyLack^®^, hence its biodegradation.

### The BP8 Metagenomic Analysis Allowed to Identify Known Additive- and PU-Degrading Enzymes and to Propose New Activities and Metabolic Pathways Involved in Biodegradation

Exploring the BP8 metagenome, genes encoding enzymes presumably involved in the degradation of the PolyLack^®^ additives were identified in several of the deconvoluted genomes. Genes for NMP degradation, similar to the ones reported for *A. denitrificans* BQ1 (Solís-González et al., 2018) were identified in the *Paracoccus* sp. BP8 genome. *Paracoccus* strains, able to utilize NMP as carbon source, have been reported (Cai et al., 2014), but the genes sustaining this capability have not been described. IP biodegradation occurs by oxidative pathways in *P. denitrificans* GH3 and *Gordonia* sp. TY-5. In these strains, IP is transformed by NAD^+^-dependent secondary ADH into acetone that is oxidized by a specific monooxygenase to produce methyl acetate, which is transformed to acetic acid and methanol (Kotani et al., 2003; Geng et al., 2015). However, the enzymes for metabolizing acetone by these reactions are not encoded in the BP8 metagenome. Instead, genes encoding the enzymes acetone carboxylase, 3-oxoacid-CoA transferase, and acetyl-CoA C-acetyltransferase were identified. These enzymes would produce acetoacetate, acetoacetyl-CoA and acetyl-CoA, respectively (Schühle and Heider, 2012) (Figure 7A and Table 3). The possibility that IP degradation occurs by transformation to acetyl-CoA, via acetone in BP8 is supported by the observation that in the *Paracoccus* sp. BP8 genome, a gene encoding an ADH (RQP05888.1), homologous to the *Gordonia* sp. TY-5 adh2, and genes encoding the acetone carboxylase subunits (RQP05866.1, RQP05867.1, RQP05889.1) are contiguously located. Adjacent to these genes, a sequence encoding a sigma-54-dependent transcriptional regulator (RQP05868.1) was observed, suggesting an operon-like organization. This presumptive IP degradative operon has not been described in any other bacteria. Degradation of 2-BE, DPGM and DPGB, the glycol ethers present in PolyLack^®^, has not been reported in bacteria. Degradation pathways for PEG and polypropylene glycol (PPG) reported in *Sphingomonads* species and *Microbacterium* (formerly *Corynebacterium*) sp. No. 7 (Kawai, 2010; Ohtsubo et al., 2015) show similar reactions where the glycols’ hydroxyl terminal groups are sequentially oxidized by specific ADHs and ALDHs to produce aldehydes, and thereafter carboxylic acids (Ohta et al., 2006; Tani et al., 2007), suggesting a widespread strategy for glycol ethers metabolism in prokaryotes. Nevertheless, few enzymes involved in scission of ether bonds, present in these compounds, have been identified in bacteria. A glycolic acid oxidase (Yamanaka and Kawai, 1991) and a glycolic acid dehydrogenase (Enokibara and Kawai, 1997) have been reported acting on PEG, although several other enzymes such as superoxide dismutase, monooxygenase, ether hydrolase, carbon-oxygen lyase, peroxidase and laccase have been suggested (Kawai, 2010). Homolog genes for specific ADHs and ALDHs were identified in the *Paracoccus* sp. BP8 genome (Table 3). Therefore, we hypothesize that degradation of 2-BE could be carried out by subsequent oxidations of the hydroxy terminal groups by PEG-DH and PEG-ALDH to produce 2-butoxyacetic acid, followed by scission of the ether bonds by glycolate oxidase or other ether scission enzymes to produce, glyoxylate and butyraldehyde (Tachibana et al., 2002; Kawai, 2010). Glyoxylate would be funneled to the glyoxylate metabolism and butyraldehyde to the butanoate metabolism (Figure 7B, left). DPGB and DPGM can be respectively degraded, by initial oxidation of the hydroxy terminal groups, to 2-butoxypropionic acid and to 2-methoxypropionic acid that has been reported as a metabolite in the degradation of DPGM by rats (Miller et al., 1986). These carboxylates can be ether-cleaved by ether scission enzymes to produce butyraldehyde and propylene glycol from DPGB and propylene glycol and formaldehyde from DPGM. Propylene glycol can be funneled to the pyruvate metabolism by lactaldehyde and lactate dehydrogenases, as suggested in *P. yeei* TT13 (Lim et al., 2018) and formaldehyde can enter to the formate metabolism where glutathione-dependent enzymes would oxidize it to formate and, subsequently, to CO_2_ (Figure 7B and Table 3). Genes encoding homologs for PEG-DH and PEG-ALDH (*pegdh* and *pegc*) from *Sphingopyxis terrae* and *S. macrogoltabida*, and for possible ether scission enzymes that could act over the aforementioned carboxylic acids, glycolate oxidase (*glcD*, *glcE*, *glcF*), dye decoloring peroxidase, 4-methoxybenzoate monooxygenase and unspecific monooxygenase were identified in *Paracoccus* sp. BP8 (see Table 3). Besides, the pathways for glyoxylate, butanoate, pyruvate and formate metabolisms as well as the TCA pathway were fully reconstructed from the BP8 metagenome (Figure 7B). Additionally, in PEG metabolism, long chains of PEG-carboxylate can be processed by acyl-CoA synthetase and glutathione-S transferase forming glutathione-conjugates (Somyoonsap et al., 2008). Although these reactions would not be needed for glycol ethers catabolism, they could be required for the degradation of long PPG moieties that are part of the PE-PU-A copolymer (Figure 3A).

By using different analytical techniques, we demonstrate that the BP8 community attacks the main functional groups of the PE-PU-A copolymer; from the more enzymatically susceptible ester bonds, present in acrylate and carbamate, to the more recalcitrant C-C from aliphatics and aromatics, C-N from urethane, and C-O-C from ether bonds of PPG (Figures 2–4). The changes in the chemical and physical properties of the polymer when incubated with BP8, and the generation of diverse degradation products, some of them potential metabolic intermediates in the degradation process, are evidences of the BP8’s degradative capability, which is sustained by the diverse xenobiotic degrading enzymes encoded in its metagenome (Table 3). Some of the biodegradation products (Figure 2B and Supplementary Table S2) seem to be the result of oxidative reactions on C-C bonds flanking TDI, MDI or the acrylates’ styrene ring (Figures 3A, 4), generating aromatic compounds containing hydroxyl, aldehydes or organic acids. Additionally, the copolymer aromatic compounds could be destabilized by monooxygenases, which introduces hydroxyl groups to the aromatic rings, and by dioxygenases that catalyzes reductive dihydroxylation, generating central intermediates that can be cleaved by dearomatizing dioxygenases producing carboxylic acids (Ladino-Orzuela et al., 2016). The enzymes for the complete benzoate metabolism are encoded in the BP8 metagenome and could account for PE-PU-A aromatic rings catabolism (Table 3). Aliphatic chains from acrylates and PPG can be metabolized by alkane 1-monooxygenases, that activate aliphatic chains by terminal or subterminal oxidations and by the activities of ADHs and ALDHs, generating compounds that can be channeled by beta-oxidation into the fatty acids metabolism (Table 3). If terminal oxidations are introduced, primary alcohols are generated and transformed into aldehydes, carboxylic acids and acyl-CoA. If subterminal oxidations of aliphatic chains occur, secondary alcohols are formed, which upon breakdown, will produce ketones and thereafter esters, which are hydrolyzed to alcohol and organic acids (Rojo, 2009). Many different esters compounds were identified in the BP8’s degradation products, suggesting that sub-terminal oxidation of alkanes could be an important route in PU metabolism (Figures 2–4). The cleavage of ester bonds by PU-esterases would produce alcohols and organic acids and the cleavage of urethane groups by carbamate-hydrolases would produce nitrogen-containing compounds and aromatic isocyanate derivatives. As we detected these degradation products by GC-MS analysis (Figure 2B, Supplementary Figure S1 and Supplementary Table S2), hydrolysis of ester and urethane bonds are accomplished during PE-PU-A degradation by BP8. The identification of several PU-esterases and carbamate hydrolases encoded in most of the BP8 genomes support this conclusion (Table 4).

### The Biodegradative Activity in BP8 Community Seems to Be Dominated by the Most Abundant Species, but Specialized Reactions Seem to Occur in Poorly Represented Species

The metabolic reactions proposed for the degradation of the additives and the PE-PU-A copolymer present in PolyLack^®^ by the BP8 community are based on the phenotypic potential encoded in its metagenome. The use of Hi-C proximity ligation-based technology allowed to define, with high confidence, what genes belong to each of the different species of BP8 (Table 3). In this community, xenobiotic degradation is a niche dominated by *Paracoccus* sp. BP8 and *Ochrobactrum intermedium* BP8.5. In their genomes, key enzymes for different steps of biodegradation are widely represented (Table 3), which must be the reason for their preponderance in the BP8 community. In addition, *Microbacteriaceae* bacterium BP8.4 genome encodes enzymes for the metabolism of aromatic compounds suggesting that metacleavage ring excision and muconate lactone formation might be functional. On the other hand, *Chryseobacterium* sp. BP8.2 and *Parapedobacter* sp. BP8.3 genomes, harbor genes encoding complementary metabolic activities for alkanes oxidation, such as hydrolysis and oxidation of linear intermediates. The finding of such a diverse genetic repertoire in the BP8 metagenome suggests a remarkable metabolic versatility, with strong hydrolytic and oxidative capabilities that can play significant roles in the degradation of diverse environmental contaminants. The abundance and distribution of these catabolic enzymes among the different members of the BP8 community, suggest syntrophic mechanisms driving community behavior. However, incomplete genome reconstruction in the deconvolution analysis, resulting in potential pathway gaps in certain genomes, cannot be ruled out, nor can the collapsing of multiple strains into a single cluster. On the other hand, although *Paracoccus* and *Ochrobactrum* are predominant in the BP8 community by far, we cannot discard that specific enzymatic activities encoded in genomes of little abundant species can be crucial for the successful performance of BP8.

### The BP8 Microbial Community as a Promising Source for Environmental Biotechnology Strategies

The present work provides deep understanding of the biodegradative activity of a landfill microbial community capable of PU and xenobiotics degradation. Results reveal the taxonomic composition of the BP8 community and its outstanding phenotypic potential, reflected in the catalytic capabilities displayed by its members to cleave different recalcitrant functional groups. This is one of the few studies integrating analytical chemistry with metagenomics for proposing metabolic pathways involved in xenobiotics biodegradation, and the first metagenomic analysis of a PU-degrading selected landfill community. Moreover, the knowledge generated about the members of the BP8 community, the potential metabolic pathways involved in PU and additives degradation, and in which species specific enzymatic reactions are carried out, could be exploited for our benefit. Some possibilities include the assembling of specific consortia for increased PU-degrading ability, the overexpression of additives- or PU-degrading proteins to be used in environmental biotechnology strategies for waste treatment, or the development of new biocatalyzers for novel industrial applications. Altogether, these features place BP8 community as a quite promising source for developing environmental biotechnology strategies contributing to mitigate anthropogenic plastics and xenobiotics pollution, for achieving better environmental quality.

## Data Availability Statement

The datasets generated for this study can be accessed from GenBank under BioProject Accession number: PRJNA488119. The microbial shotgun “*de novo*” assembly and the log file for the assembly run done with metaSPAdes software were deposited at Mendeley: https://data.mendeley.com/datasets/bkwf2xhytj/1.

## Author Contributions

IG, AS-R, MB, MV-S, IL, and HL-T contributed to conception and design of the study. IG and MB conducted the analytical techniques and interpreted the results along with MC-G and HL-T. IL, MP, and SS performed metagenomics experiments. AS-R and HL-T analyzed the metagenomic data. IG, AS-R, MB, MV-S, IL, MC-G, and HL-T wrote the manuscript. All authors contributed to manuscript revision, read and approved the submitted version.

## Conflict of Interest

IL, MP, and SS are employees and shareholders of Phase Genomics Inc., a company commercializing proximity ligation technology. The remaining authors declare that the research was conducted in the absence of any commercial or financial relationships that could be construed as a potential conflict of interest.

## Footnotes

1 http://phylogeny.lirmm.fr/phylo_cgi/one_task.cgi?task_type=gblocks
