# Supplementary material for: Degradation of Recalcitrant Polyurethane and Xenobiotic Additives by a Selected Landfill Microbial Community and Its Biodegradative Potential Revealed by Proximity Ligation-Based Metagenomic Analysis
Source: Front Microbiol. 2020 Jan 22;10:2986. doi: 10.3389/fmicb.2019.02986 (PMC6987047; doi:10.3389/fmicb.2019.02986)
Supplement: Supplementary file 1 [file Data_Sheet_1.pdf]

Supplementary Table S1. PolyLack additives consumption during cultivation of BP8 community.

*Additive (%)	Non-inoculated	Culture time (days)							
		1	2	3	4	5	10	15	20
1. 2-butoxyethanol	100	22.0	19.2	7.5	3.5	0	0	0	0
2. Dipropylene glycol methyl ether	100	16.1	14.1	15.0	15.5	17.6	16.0	16.1	16
3. N-methyl-2-pyrrolidone	100	0	0	0	0	0	0	0	0
4. Dipropylene glycol butyl ether	100	27.2	26.5	27.7	26.1	26.3	28	27.1	30

*Standard curves set with pure compounds were used for quantification of additives. Additives concentration in non-inoculated samples of MM-PolyLack was considered 100%. Numbers of the compounds represent the peaks in the chromatograms shown in Supplementary Figure S1.

Supplementary Table S2. Biodegradation products identified during the cultivation of the BP8 community in MM-Polylack.

*Biodegradation products (area)	Culture time (days)								
	Non-inoculated	1	2	3	4	5	10	15	20
**Aromatics**									
5. 2,4-Toluene diisocyanate derivative	396284	210323	330578	299568	207044	356398	331245	217019	249605
6. Methylene diphenyl diisocyanate derivative	287793	220793	54595	50936	55191	108873	136280	131777	116545
7. 3,5-Di-*tert*-butylphenol	0	0	0	35787	12051	58975	415594	140426	130889
8. 2-Benzyl-5-oxocyclohexanecarboxylic acid	0	0	22217	27656	26630	46695	68912	31559	47082
9. α-2-Propenylbenzeneacetaldehyde	0	0	16897	35787	50086	46693	305653	116986	269677
10. 4-Methylcinnamic acid ester	0	0	0	47059	50393	72610	183476	76501	70074
**Nitrogen containing**									
11. *N,N*-Diethylhydroxylamine	0	8231	5146	8267	5177	18239	3494	18216	15901
12. 2-Hydroxybutanoic acid hydrazide	0	0	0	4802	0	8007	49400	15322	13816
13. Stearic acid hydrazide	0	0	0	13367	8932	0	0	23822	9188
14. *O*-Decylhydroxylamine	0	0	0	0	28876	62425	302484	78756	73626
**Ethers**									
15. Propylene glycol butyl ether	0	35235	91892	79984	55877	70096	274909	58458	357803
16. 1,2,3-Propanetriol, 1-indol-4-yl- ether	0	0	0	23665	17955	19367	195926	59607	67136
17. Oxalic acid di-*sec*-butyl ester	0	0	27629	52302	49504	73678	186661	33920	47228
18. 1-Ethyldecyl acetate	0	16872	15161	15074	18018	31218	134304	66141	33494
19. Tetrahydrolinalyl acetate	0	13580	21962	16917	18859	30560	138363	47297	30864
20. 1-Methyldodecyl acetate	0	24505	29517	21388	22637	71030	271827	154162	150944
21. 2-Ethylundecyl acetate	0	29597	34345	28447	50883	115466	274808	168235	172285
22. 2-Palmityl acetate	0	0	0	0	124898	254356	419171	264126	173680
23. 1-Ethyltridecyl acetate	0	16932	0	14688	22987	16672	183159	61590	45659
24. Lauric acid methyl ester	0	15982	0	0	0	42782	216994	47712	2583
**Aliphatics**									
25. 2,4-Dimethyl-1-heptene	80333	12324	41466	23238	20216	34279	108717	43372	44517
26. 2,6,10-Trimethyltetradecane	0	0	0	14594	146501	48409	136754	37755	48979
**Alcohols**									
27. 4-Hexadecanol	0	17016	26631	27332	12921	72491	228358	186127	134788
28. 2-Hexyl-1-decanol	0	15798	11251	10553	15751	37688	185452	68618	35932
**Organic acids**									
29. 3-Hydroxy-3-methylbutyric acid	0	0	0	10184	12020	4998	65496	35579	14114

*Numbers of the compounds represent the peaks in the chromatograms shown in Supplementary Figure S1.

Supplementary Table S3. Effects of BP8 biodegradative activity on the PE-PU-A copolymer analyzed by Differential Scanning Calorimetry.

Culture time (days)	Tg (°C)	Tm-I (°C)	Tm-II (°C)	Tm-III (°C)	Tc (°C)
Non-inoculated	50.2	70.0	210.6	398.1	459.6
5	39.5	68.8	211.0	408.7	478.2
10	46.0	68.0	210.1	407.9	480.2
15	38.1	70.5	211.1	393.2	479.9
20	46.2	78.8	213.9	392.5	476.2

Tg = glass transition temperature; Tm = melting temperature; Tc = crystallization temperature.

Supplementary Table S4. [a]Phylogenetic relatedness of the bacterial species from the BP8 community identified by Hi-C metagenome deconvolution.

Clusters/ Clasification/ GeneBank Acc. Num.	Organism	Assembly	Genome size (Mb)	GC content (%)	[a]ANI value (%)	Proteins encoded in the genomes
Cluster 1/ *Paracoccus* sp. BP8 GCA_003852815.1	*Paracoccus* sp. J39	GCA_000518925.1	4.42837	68.08	98.73	4131-4993
	Paracoccus denitrificans	GCA_000203895.1	5.23619	66.78	90.24	
	Paracoccus aminovorans	GCA_001546115.1	4.58940	67.50	85.59	
	Paracoccus halophilus	GCA_900111785.1	4.00871	65.20	82.17	
	Paracoccus yeei	GCA_002073635.2	4.82967	67.08	81.95	
Cluster 2/ *Chryseobacterium* sp. BP8.2 GCA_003852805.1	*Chryseobacterium koreense*	GCA_001045435.1	3.15420	40.10	69.38	3000-4810
	Chryseobacterium camelliae	GCA_002770595.1	4.37635	41.80	68.86	
	Chryseobacterium luteum	GCA_000737785.1	4.71855	37.30	68.70	
	Chryseobacterium sp. 52	GCA_002754245.1	5.29882	37.00	68.49	
	Chryseobacterium antarcticum	GCA_000729985.1	3.12366	36.10	68.10	
Cluster 3/ *Parapedobacter* sp. BP8.3 GCA_003852785.1	*Parapedobacter indicus*	GCA_900113765.1	6.15523	48.00	80.23	3796-4949
	Parapedobacter koreensis	GCA_900109365.1	5.54776	48.20	74.24	
	Parapedobacter luteus	GCA_900168055.1	4.82992	49.30	73.66	
	Parapedobacter composti	GCA_900112315.1	4.62202	50.00	73.32	
Cluster 4/ Microbacteriaceae bacterium BP8.4 GCA_003852775.1	*Micrococcales bacterium* 72-143	GCA_001898835.1	3.33048	71.60	85.07	2535-3047
	[b]*Leifsonia* sp. Leaf336	GCA_001423695.1	4.15779	69.60	74.40	
	[b]*Microcella alkaliphila*	GCA_002355395.1	2.70284	68.40	74.10	
	[b]*Plantibacter* sp. H53	GCA_001650455.1	4.01278	69.40	73.98	
	[b]*Herbiconiux* sp. YR403	GCA_000799285.1	3.60404	62.00	71.66	
	[c]*Arthrobacter cupressi*	GCA_900099975.1	4.0588	67.00	70.82	
Cluster 5/ *Ochrobactrum intermedium* BP8.5 GCA_003852825.1	*Ochrobactrum intermedium* LMG 3301	GCA_000182645.1	4.72539	57.70	98.48	3645-4502
	Ochrobactrum anthropi	GCA_000017405.1	5.20578	56.15	87.48	
	Ochrobactrum oryzae OA447	GCA_002943495.1	4.46700	56.20	87.19	
	Brucella ceti	GCA_000590795.1	3.27803	57.27	82.03	
	Ochrobactrum sp. P6BS-III	GCA_002016635.1	5.25313	56.00	81.19	

[a] Phylogenetic relatedness was calculated based on ANI value with OrthoANIu algorithm**.** ANI values higher than 95% indicate that the compared genomes are the same species (Yoon S-H, Ha S, Lim J, Kwon S, Chun J. A large-scale evaluation of algorithms to calculate average nucleotide identity. Antonie Van Leeuwenhoek. 2017; 110:1281-6).

[b] Members of *Microbacteriaceae* family, order Micrococcales.

[c] Member of *Micrococcaceae* family, order Micrococcales.
