# Supplementary material for: Degradation of Recalcitrant Polyurethane and Xenobiotic Additives by a Selected Landfill Microbial Community and Its Biodegradative Potential Revealed by Proximity Ligation-Based Metagenomic Analysis
Source: Front Microbiol. 2020 Jan 22;10:2986. doi: 10.3389/fmicb.2019.02986 (PMC6987047; doi:10.3389/fmicb.2019.02986)
Supplement: Supplementary file 2 [file Data_Sheet_2.PDF]

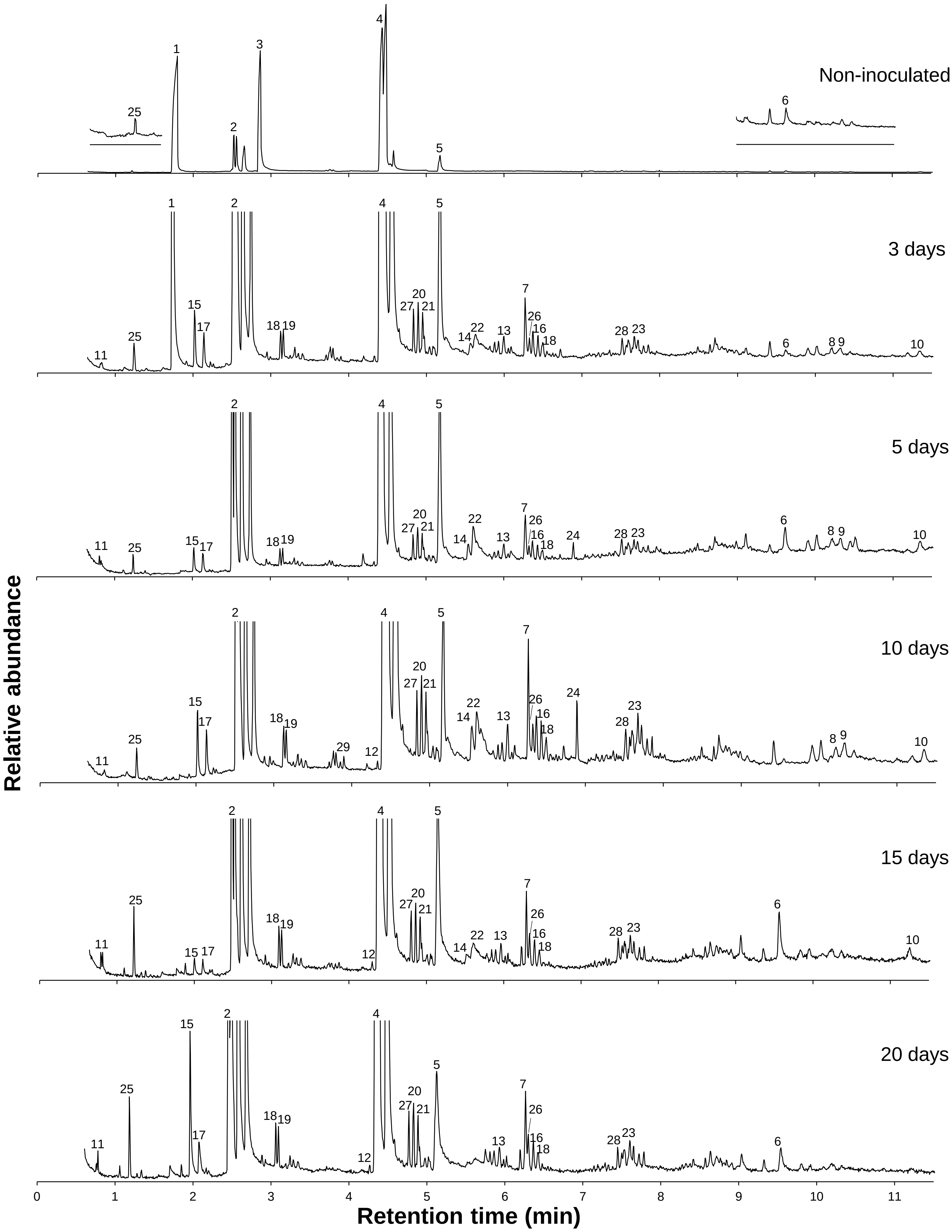

Supplementary Figure S1. Chromatograms of cell-free supernatants from BP8 cultures grown in MM-PolyLack. Numbered signals designate compounds identified by mass spectrometry listed in Figure 2. Chromatogram of non-inoculated sample is at original scale, whereas the other chromatograms are at larger scale.

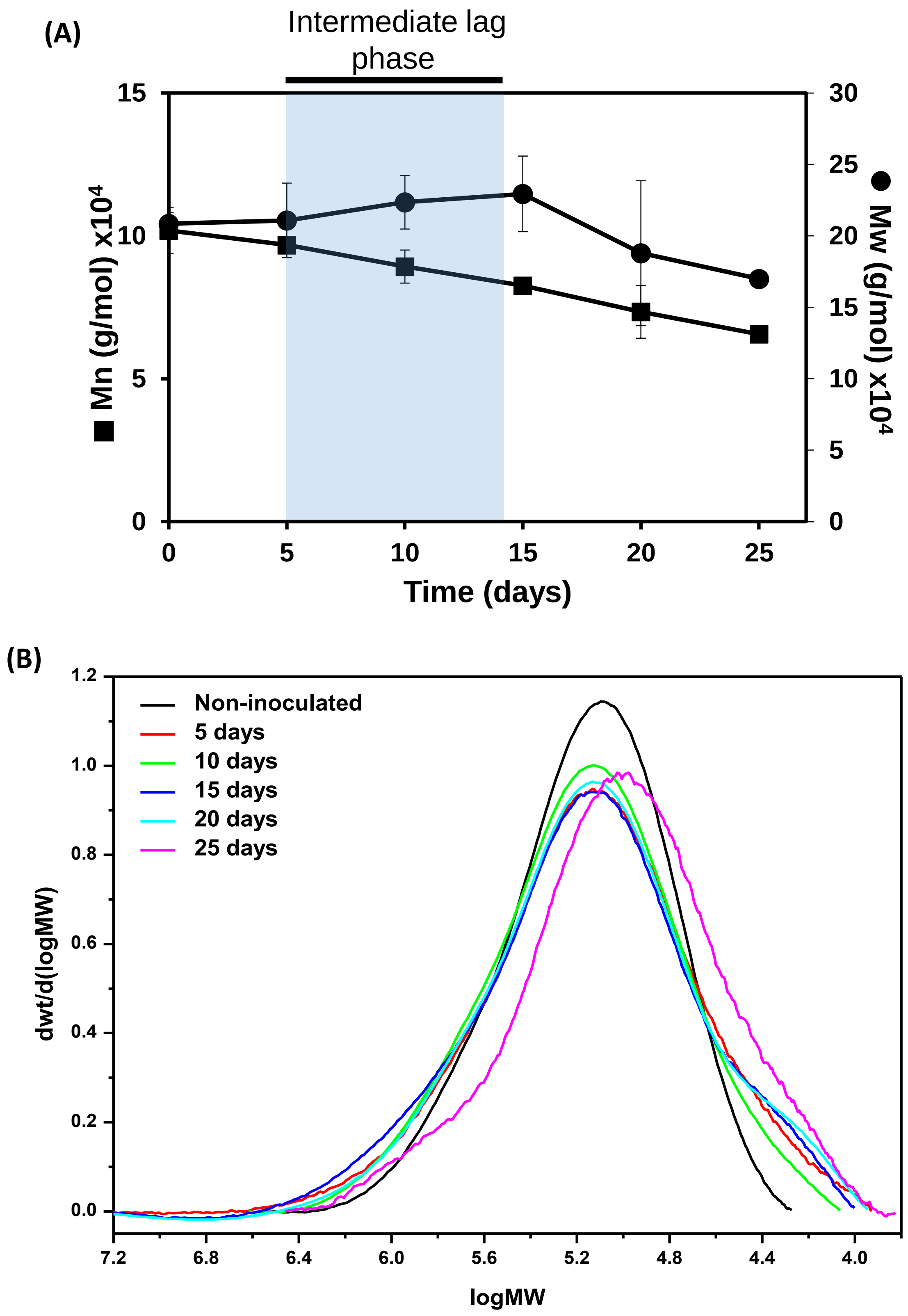

Supplementary Figure S2. (A) Number-average molecular weight (*Mn*) and weight-average molecular weight (*Mw*) and (B) Molecular weight distribution (MWD) of the PE-PU-A copolymer present in PolyLack, during the cultivation with the BP8 community. In (A), the intermediate lag phase is shown (blue rectangle) for analysis.
